# Patterns of brown bear (*Ursus arctos*) visits to human settlements provide insights for human–wildlife coexistence

**DOI:** 10.1038/s41598-026-47443-4

**Published:** 2026-05-17

**Authors:** Manuel Díaz-Fernández, Javier Naves, Miguel de Gabriel Hernando, Eloy Revilla

**Affiliations:** 1 Fundación Oso de Asturias, Ctra. General, s/n, Proaza, Asturias, 33114 Spain; 2 Department of Conservation Biology and Global Change, Estación Biológica de Doñana, Consejo Superior de Investigaciones Científicas (EBD- CSIC), Calle Américo Vespucio s⁄n, Sevilla, 41092 Spain; 3 Department of Biodiversity and Environmental Management, Faculty of Biological and Environmental Sciences, Universidad de León, León, Spain

**Keywords:** Brown bear (*Ursus arctos*), Human-wildlife interactions, Coexistence, Management, Population recovery, Ecology, Ecology, Zoology

## Abstract

Negative interactions between humans and large carnivores represent one of the main challenges for the conservation of these species due to the social conflict they can generate. In the case of brown bears (*Ursus arctos*), these interactions include visits of bears to human settlements, a type of event increasingly reported in recent years and often associated with conflicts. In this study, we collected 73 events of bear visits to human settlements between 2009 and 2021 in the Cantabrian population. These events were mainly caused by young individuals, occurring mostly at night and during the summer, when bears were primarily attracted by fruit trees available in human settlements. The affected settlements were located in areas of high habitat quality, close to breeding cores, and with a high intensity of bear damage. Within these areas, human settlements with bear visits differed from nearby ones by having a larger perimeter, a shorter distance to forest patches, and greater surrounding terrain ruggedness. Our study provides a basis for understanding the patterns and drivers of these particular human-large carnivore interactions and offers valuable insights to update and improve management strategies aimed at preventing such events, mitigating associated conflicts and promoting successful coexistence.

**Supplementary Information:**

The online version contains supplementary material available at 10.1038/s41598-026-47443-4.

## Introduction

In recent decades, several populations of large carnivores have shown encouraging signs of demographic recovery in different parts of the world^1^. The presence of large carnivores has positive ecological effects, as they play a key role in ecosystem functioning^2^ and they often act as a flagship species that can provide social and economic benefits for local communities^3^. However, the recovery of large carnivores sometimes implies complex challenges due to social conflicts caused by negative human-carnivore interactions^4^. In a context where a significant percentage of the Earth’s surface has been altered by human activities^5^, an increase of these interactions is expected^6–8^ which could jeopardize coexistence with large carnivores and, ultimately, the conservation of these species.

In recent decades, the brown bear (*Ursus arctos*) has experienced a significant increase in abundance and distribution in some areas of Europe^1,9^. This recovery, occurring within a densely populated area increasingly occupied by human activities^10^, has intensified negative human-bear interactions, not only raising human safety concerns but also undermining large carnivore conservation efforts^11–14^. Despite their general avoidance of human-dominated landscapes^15–17^, bears are increasingly forced to share habitats with humans due to the lack of optimal habitats and undisturbed areas^16,18^. Some studies highlight how bears are attracted to anthropogenic food sources in campsites or settlements in North America^19,20^, Japan^21^ and Europe^13,14,22^. The proximity of recovering bear populations to human activities^7^ increases the risk of these interactions^23,24^. The increasing and continuous exposure to interactions with humans that do not result in direct negative consequences may lead some bears to undergo a process of habituation to human stimuli to varying degrees^25,26^. Similarly, some individuals learn to associate humans (infrastructure, activities, or presence) with a reward in the form of food (livestock, crops, beehives, garbage, etc.), undergoing a process of food conditioning to anthropogenic food resources^25,27–29^.

In the Cantabrian Mountains, the brown bear population has been increasing in abundance and distribution since the end of the 20th century^30,31^, when its numbers dropped to fewer than 100 individuals, separated into two isolated subpopulations^32^. Nowadays, the population has around 320–370 individuals^30^ distributed around 17.000 km^2 [31]^. During the recovery process, the species has been associated with a broadly positive social perception across territories and stakeholders^33^, linked to its role as a flagship species for conservation and to perceived socioeconomic benefits^34^. However, as the population recovered and recolonized new areas, other negative human-bear interactions such as property damage, also emerged, particularly in zones with less experience in preventive measures^35–37^. Furthermore, reports of bears approaching or entering human settlements appear to become more frequent, potentially reflecting increasing public and media attention to this issue. These events may also heighten perceived risks to personal safety among residents, which could contribute to more negative attitudes and reduce tolerance among part of the society, potentially generating conflict scenarios^38,39^.

Despite the growing media coverage, the associated social repercussions, and the management actions currently implemented to address bear visits to human settlements, these events have not yet been described or analysed in scientific studies in this population. Therefore, the main aims of this study are: (1) to describe the spatiotemporal patterns and main characteristics of bear visits to human settlements; (2) to identify the landscape and local-scale factors driving their occurrence in order to predict the probability of such visits; and (3) to provide management guidelines for assessing and mitigating the risks associated with these visits. We hypothesized that bear visits to human settlements are not random, but rather respond to a combination of ecological and spatial factors. Specifically, we expected that these visits would occur more frequently in areas of high habitat quality and in settlements located closer to forest patches and food resources, such as fruit trees. We also hypothesized that the probability of visits within settlements increases in places situated near breeding areas and in landscapes with a greater incidence of bear damage, reflecting the influence of both environmental and behavioral factors on these visits.

## Methods

### Study area and bear population

The Cantabrian Mountains run parallel to the northern coast of the Iberian Peninsula, spanning approximately 300 km from west to east. The landscape is predominantly characterized by mixed forests of oak (*Quercus petraea*,* Q. pyrenaica*,* Q. rotundifolia*), beech (*Fagus sylvatica*), birch (*Betula celtiberica*), and chestnut (*Castanea sativa*). The areas above the tree line are dominated by some species of subalpine shrubland: *Juniperus communis*, *Vaccinium uliginosum*, *V. myrtillus* and *Arctostaphylos uva-ursi*. The Cantabrian brown bear population is distributed in two connected subpopulations occupying around 17,000 km^2 31^ with around 250 individuals in the western subpopulation and 120 bears in the eastern subpopulation^40^. The species is distributed throughout the four different administrative regions: Asturias, Cantabria, Castilla y León and Galicia. The human population is mainly distributed in small settlements with an average human density of 10.9 people/km^2^ for the western part and 4.9 for the eastern part^12^.

### Data on bear visits in human settlements

We officially requested all available official records of bear visits to human settlements until 2021, from the four regional administrations within the Cantabrian brown bear range (Castilla y León, Asturias, Cantabria and Galicia) (Supplementary Fig. 1). No data were available from Galicia, as no events of bear visits were reported during the study period. In Spain, there is an intervention protocol for handling human–bear conflict situations, including approaches to human settlements[^42^]. Each regional administration manages brown bear protection and monitoring through specialized ranger patrols. However, there is no unified system for documenting such events, and the information provided was not always complete. To complement the official datasets, we collaborated with non-governmental organizations dedicated to the conservation of the Cantabrian brown bear. Specifically, we obtained additional records from the Asturias Brown Bear Foundation (Fundación Oso de Asturias, FOA) for Asturias and the Brown Bear Foundation (Fundación Oso Pardo, FOP) for Castilla y León and Cantabria. Finally, we conducted a digital press search using the terms “bear”, “village”, “Asturias”, “León”, “Palencia”, “Cantabria” and “Galicia” to identify further events of bear visits to human settlements that were reported in the media but not recorded by the official sources. To ensure data reliability, these media reports were only included when accompanied by multimedia evidence (i.e., videos or photographs) that unequivocally confirmed both the bear’s presence and its location within the specific settlement mentioned, allowing for visual cross-verification of the site.

Due to the non-standardized nature of the initial records provided by regional administrations and other sources, we applied a filtering and validation process. Following this, we defined a *bear visit* as any documented presence of a brown bear within the boundaries of a human settlement. These boundaries were delineated using the official BTN100 dataset (1:100000 scale) from the Spanish National Geographic Institute (IGN), which provides a standardized delimitation of the consolidated urban matrix, encompassing the built-up area (houses) and immediate urban lands, such as private gardens or orchards. By using this delimitation and cartographic resolution, we ensured that spatial metrics (e.g., area and perimeter) reflected consolidated human-occupied zones rather than isolated buildings or small agricultural sheds that are not part of the urban settlement matrix. While this may omit some very small, dispersed dwellings, it provides a consistent and reproducible framework for analyzing bear visits across the entire study region. To ensure data independence, each record was treated as a separate event if it occurred in a different human settlement or on a different date. When multiple bear visits were reported within the same human settlement or close, they were treated as distinct events only when expert reports from rangers or NGO technicians provided evidence of different individuals (e.g., based on phenotypic traits such as size, age class, or the presence of females with cubs vs. solitary bears).

To describe the spatiotemporal patterns and characteristics of bear visits, we conducted field verifications in settlements with reported events, accompanied by wildlife rangers. For each event, we recorded spatiotemporal details, including the coordinates of the location where bears were observed or evidence of their presence was detected (scats, footprints, damage, etc.), the time of day the event occurred (categorized as day, night, or twilight) and the duration of the events (number of days). Individual-level characteristics, including group size, sex, and age class (subadult, adult, or family group), were assigned based on professional reports from wildlife rangers and NGO technicians. We also documented behavioral traits, including feeding signs and the type of trophic attractant, evidence of material damage, and interactions involving domestic animals and humans. We categorized interactions with humans based on reports from rangers and NGO technicians according to the definitions proposed by Hopkins^28^. We distinguished between “human-bear interaction”, defined as an occurrence where both a person and a bear were mutually aware of each other (e.g., through visual encounters or auditory stimuli such as vocalizations), and “tolerance of human presence”, a behavioural state in which a bear does not take evasive or aggressive action when in the presence of humans. Finally, we also collected information on ranger interventions in these cases, the type of method used and the result obtained (Supplementary Data S1).

### Analyses

To identify the landscape and local-scale factors driving bear visits to human settlements we used data from 44 human settlements where such events were recorded between 2009 and 2021. These settlements were compared with others located within the Cantabrian brown bear distribution range that had no recorded visits, while acknowledging that some nocturnal or fleeting presence may have gone undetected (false negatives). We selected the period 2013–2021[^31^] to define the bear distribution range because all events, except one recorded prior to 2012, were recorded within this time frame. The analysis was structured into two scales to identify the factors influencing the likelihood of a human settlement being visited by a bear.

**Fig. 1:**
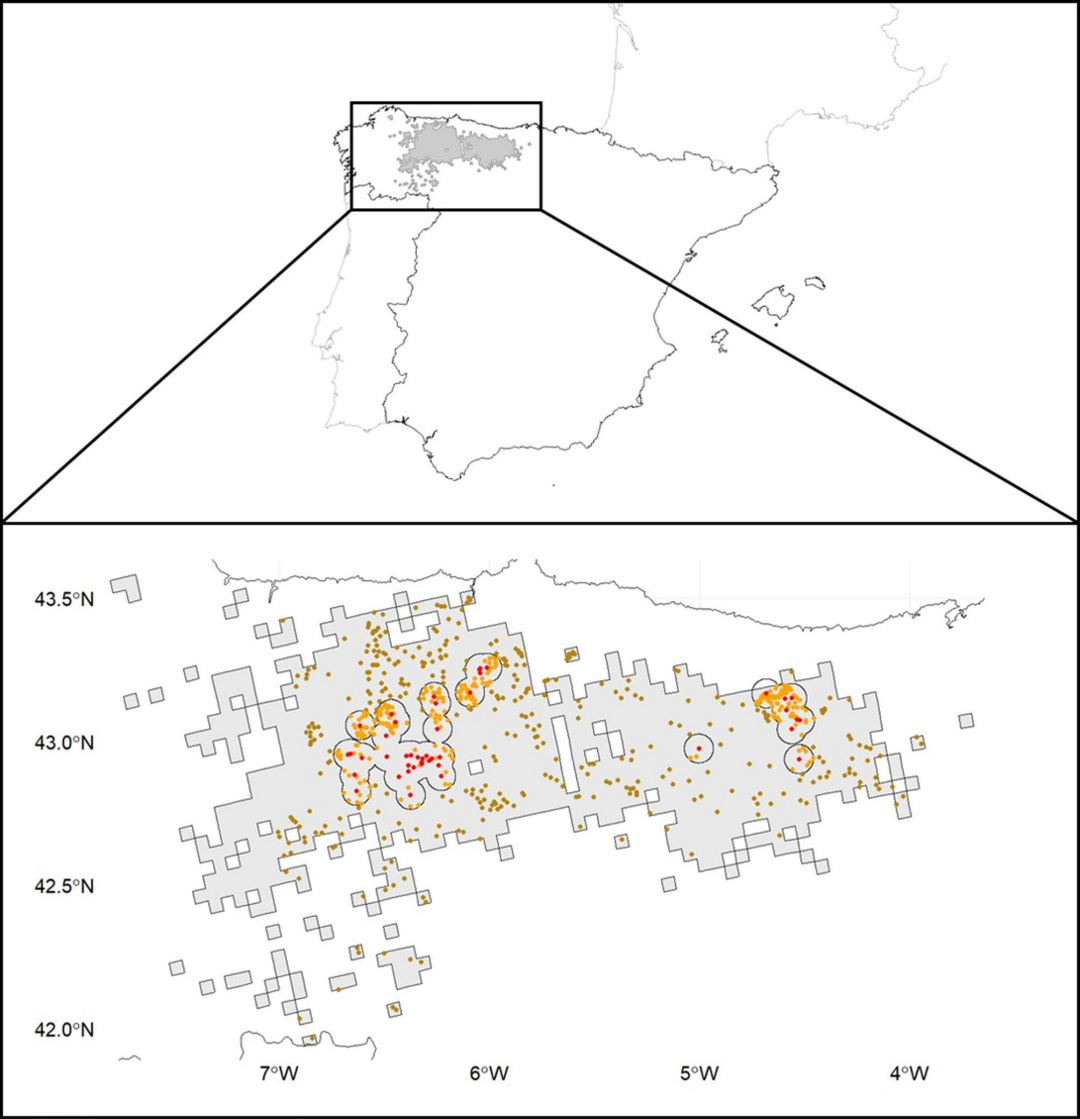
Study area location. The upper panel shows the distribution of the Cantabrian brown bear during the period 2013-2021[^31^]. The lower panel provides the spatial distribution of human settlements with bear visit events (red) along with a 5.6 km buffer around them, based on the minimum home range of a brown bear in Europe. Human settlements without recorded bear visits within the home range used for the local-scale analysis are shown in orange, while human settlements without recorded bear visits used for the landscape-scale analysis are shown in gold. Note that some of the human settlements without recorded bear visits at the landscape-scale matched with some human settlements without recorded bear visits occurrences at the local-scale. The maps were generated using R version 4.3.1 available at https://cran.rstudio.com/ and administrative boundaries were obtained from Eurostat GISCO. © EuroGeographics for the administrative boundaries.

At the landscape scale we focused on the characteristics surrounding the human settlements that could increase their probability of being visited by bears. We compared human settlements with bear visits events to those without recorded bear visits, located within the entire Cantabrian brown bear distribution range (Fig. 1). As the number of human settlements without recorded bear visit events greatly exceeded those with events (1641 vs. 44), we randomly selected a subset of 440 human settlements without recorded bear visits (ten times the number of human settlements with events). We used generalized linear models (GLMs) with the “stats” package in R (version 4.3.1), using a binomial response variable to indicate bear visit in a human settlement (presence = 1; random absence = 0). To address class imbalance, we applied the weights parameter in the GLM function, assigning a weight of 0.1 to observations with case = 0 (440) and 1 to those with case = 1 (44).

At the local scale we focused on the factors that might influence a bear’s decision to enter a specific human settlement over others within its range. For this, we compared human settlements with bear visits events to human settlements without recorded bear visits that were within the minimum annual home range of a bear in Europe that is 25 km^2^ (see Table S2 in the supplementary material of Hertel et al., 2025[^75^]). The radius of a buffer covering this area is 2.8 km. However, we accounted for the fact that a human settlement where bear visits had been recorded may not be at the center of the home range but could instead be near its edge. Therefore, we opted to use a 5.6 km buffer around each human settlement with at least one recorded bear visit and included all human settlements without recorded bear visits located within this radius. We removed duplicate human settlements due to the overlap of some buffers, resulting in a total of 235 human settlements without recorded bear visit events (Fig. 1). We constructed GLMs with a binomial response variable (presence = 1; absence = 0).

At the landscape scale, we considered that the variables that explain the probability of bear visit to a human settlement are those that relate the location of the human settlement to areas with higher likelihood of bear presence. We used habitat-related variables, such as the habitat quality indices developed by Martin et al., (2012)^18^(natural index, *NAT*, and human index, *HUM*), which include a series of factors related to the species’ survival and reproduction. We also included a variable linking the human settlement’s location relative to the bear population, specifically the distance from each human settlement to the centroid of the nearest breeding core (*DistBC*). Here, breeding cores refer to the areas occupied by females with cubs (family groups), defined on the basis of direct observations collected by the wildlife administration during the annual censuses of females with cubs. Additionally, we incorporated the number of bear damage claims in the 5 × 5 km grid cell where the human settlement is located for the 2012–2018 period (*N-Dam*). This variable is associated with the availability of anthropogenic food resources but also reflects the presence of the species and individual behavior. Finally, we included a variable that explains the spatial relationship between human settlements with bear visit events, measured as the distance to the nearest human settlement with a bear visit event (*DistClosest*) (Table 1). We log transformed the distance to the nearest breeding core and the distance to the nearest human settlement with b to reduce skewness.

At the local scale, we used predictors related to the accessibility of a human settlement to a bear in the surrounding environment. These included habitat variables such as the distance from the human settlement edge to the nearest forest patch (*DistForest*), the nearest shrub patch (*DistShrub*), the nearest crop land (*DistCrops*) and the average terrain ruggedness index (*Ruggedness*) within a 1000-meter radius around the human settlement. Additionally, we considered human settlement-specific characteristics, including perimeter (*Perimeter*) and population density (number of inhabitants/area; *PopDensity*). We used this density metric as a proxy for the intensity of infrastructure and anthropogenic resources (e.g., houses, gardens, and orchards) which remain constant regardless of seasonal population fluctuations. Finally, we incorporated a variable related to the spatial clustering of human settlements with and without recorded bear visits: the number of total human settlements within a 5.6 km buffer (*Settlements*) (Table 1). We log transformed all the distance variables, the perimeter, and the population density to reduce skewness.

**Table 1:** List and description of variables used in the generalized linear models to analyze the probability of bear visit in human settlements.

Landscape scale	
Predictors	Description
*NAT*	Natural habitat index (Martin et al. 2012) of the 5 × 5 km grid in which the human settlement is located
*HUM*	Human habitat index (Martin et al. 2012) of the 5 × 5 km grid in which the human settlement is located
*DistBC*	Distance of each human settlement to the centroid of the breeding core of the subpopulation to which it corresponds
*DistClosest*	Distance of each human settlement from the nearest human settlement with a bear visit event
*N-Dam*	Total number of damages claims in the grid where the human settlement is located during the period 2012–2018
**Local scale**	
**Predictors**	**Description**
*Perimeter*	Human settlement perimeter
*PopDensity*	Human settlement population density (Human population/Area)
*Settlements*	Number of human settlements with and without recorded bear visits within a 5.6 km buffer around each human settlement
*DistForest*	Distance from the edge of the human settlement to the nearest forest patch (Corine Land Cover 2018; Codes: 311,312,313)
*DistShrub*	Distance from the edge of the human settlement to the nearest shrub forest patch (Corine Land Cover 2018; Codes: 322, 324)
*DistCrop*	Distance from the edge of the human settlement to the nearest crop land (Corine Land Cover 2018; Codes: 242,243)
*Ruggedness*	Average terrain ruggedness index in the 1 km buffer around the human settlement based on the 200 m digital elevation model

For both analyses we constructed all the models with all the possible combinations of variables using the function dredge of the “MuMIn” library, ordering them based on the Akaike Information Criterion (AICc) and selecting those models that had ΔAICc < 2. We finally performed a model average to extract the coefficients, std.errors, and p-values of each of the variables in the models in which they were included. The correlation and Variance Inflation Factor (VIF) of all variables included in the models were assessed (Supplementary Table 1; Supplementary Fig. 2; Supplementary Fig. 3).

## Results

### Spatiotemporal patterns and main characteristics of bear visits

We compiled 73 cases of brown bear visits to human settlements in the Cantabrian population during the period 2009–2021. We obtained information on cases from three regions with brown bear presence: Asturias, Cantabria, and Castilla y León. In Galicia, according to official reports, there were no records of bear visits in human settlements. Altogether, we identified 44 human settlements with documented cases of brown bear visits: 35 human settlements reported only a single case, while 9 human settlements experienced multiple cases. The settlements with the highest number of cases registered 8, 7, and 6 events, respectively (Supplementary Table 2). The largest proportion of these cases (86%) occurred in the western subpopulation of the Cantabrian brown bear population, in the regions of Castilla y León and Asturias, with 63 cases in 35 human settlements. Human settlements with recurrent cases were primarily concentrated in this area as well. In the eastern subpopulation we documented 10 cases in 9 human settlements (Supplementary Table 3; Fig. 1).

**Fig. 2:**
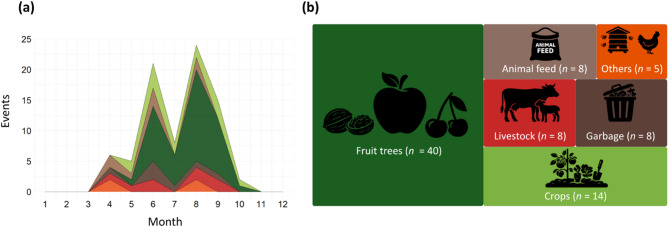
(**a**) Monthly distribution of bear visits events to human settlements showing the proportion of trophic attractants involved; (**b**) Tree map showing the number of events in which each type of trophic attractant was reported. The total number (83) exceeds the number of events (73) because multiple attractants were often reported in a single case.

The majority of the recorded events (82%) occurred during the summer months, specifically between June and September. In contrast, events were much less frequent in spring or autumn, and no cases were documented during the winter season (Fig. 2a). Feeding behavior on some trophic attractants was documented in 86% of the cases. Fruit trees were the most common trophic attractant, identified in 40 of the 73 cases (55%). Other attractants were crops in 14 cases, sheep in 8 cases, garbage in 8 cases, and different types of domestic animal feed in 8 cases. Less frequently, attractants such as chickens, grass, and beehives were recorded, collectively accounting for 5 cases. In 22 cases, multiple trophic attractants were involved, with a maximum of three different resource damaged in two of these cases (Supplementary Table 3; Fig. 2b).

**Fig. 3:**
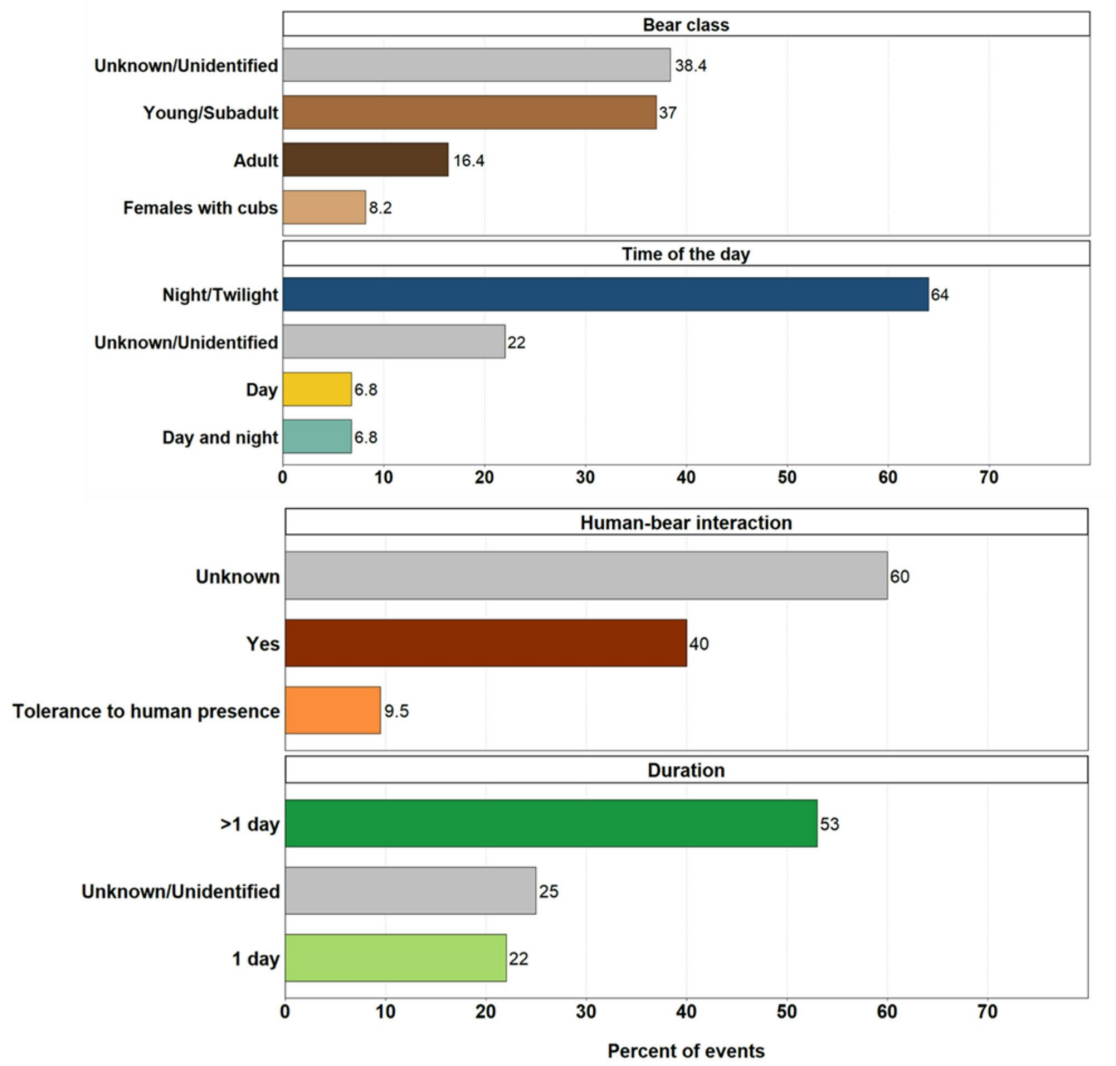
Percentage distribution of bear visits events in human settlement: (**a**) Distribution by age–sex class, showing the proportion of events involving young/subadult bears, adults, females with cubs, and unidentified individuals; (**b**) Distribution by time of day, indicating the proportion of events occurring at night/twilight, during daylight hours, both day and night, or at unknown times; (**c**) Proportion of events involving direct human–bear interactions, indicating whether any interaction occurred and, when confirmed, the percentage of cases in which bears showed tolerance of nearby human presence; (**d**) Duration of the events, showing whether bears remained near the settlement for one day, more than one day, or an undetermined period.

The bears involved were predominantly young or subadult individuals (37%), followed by adult bears (16.4%), and females with cubs (8.2%). In 38.4% of the events, insufficient data prevented precise classification of the bear sex and age class, and these were categorized as “Unknown/Unidentified” (Supplementary Table 3; Fig. 3a).

Regarding the time of day, 64% of the events took place during the nights or twilight hours. However, we identified approximately five cases where events occurred exclusively during daylight hours, and an additional five cases in which bears were observed both during the day and at night. Notably, 16 records lacked clear information to determine the specific time of the visit (Supplementary Table 3; Fig. 3b). In 39 cases (53%), the bear was reported to be present in the human settlement for more than one day. In 16 cases, the reported duration was only one day. For 18 cases, no information on the duration of the events was available (Supplementary Table 3; Fig. 3d). Additionally, of the 73 recorded cases, there was evidence of human-bear interactions (e.g., encounters, vocalizations) in 29 cases (39.7%). Notably, tolerance of human presence by the bear could be confirmed in just 7 of these cases according to the field rangers (Supplementary Table 3; Fig. 3c).

Of the 73 cases recorded, the administrations reported interventions in 26 instances. In 16 of these cases, some form of hazing^41^ was employed, including vocalizations, firecrackers, or rubber bullets. In five cases, the actions were limited to monitoring efforts aimed at locating the individual and managing human activity to prevent interactions. Preventive measures, such as the installation of electric fences or livestock enclosures, were implemented in three cases. Lastly, there were two cases in which interventions were confirmed, although the specific actions taken remain unknown (Supplementary Table 3).

### Landscape and local-scale factors driving bear visits

At the landscape scale, the probability of a human settlement having an event of bear visit increases significantly in those areas with higher values of the habitat quality index for bears (*NAT*) and with a higher amount of damage during the period between 2012 and 2018. The probability decreases significantly with increasing distance between the human settlement and the breeding core of the corresponding subpopulation. We also observed that the probability of a bear visit increases with decreasing distance to the nearest human settlement with recorded bear visits (Table 2; Supplementary Table 4).

At the local scale, we observed that the probability of a bear visit in a human settlement increases in human settlements with a larger perimeter, with a higher terrain ruggedness index within a 1 km buffer, and closer to forest (Table 2; Supplementary Table 5).

**Table 2:** Model-averaged (conditional average) estimates, std. errors, adjusted SE, z values and significance (Pr(>|z|)) for the variables included in the best generalized linear regression models (ΔAICc < 2) to explain the probability of bear visit at the landscape and local scale. See Table 1 for definitions of all predictor variables.

Landscape scale						
Variable	Estimate	Std. Error	Adjusted SE	z value	Pr(>|z|)	
*Intercept*	*-1.874*	*0.576*	*0.578*	*3.244*	*0.001*	**
DistBC	-0.989	0.396	0.397	2.491	0.013	*
NAT	1.306	0.626	0.628	2.081	0.037	*
N-Dam	0.731	0.383	0.383	1.907	0.056	.
DistClosest	-1.172	0.426	0.427	2.748	0.006	**
HUM	-0.201	0.577	0.578	0.349	0.727	
**Local scale**						
**Variable**	**Estimate**	**Std. Error**	**Adjusted SE**	**z value**	**Pr(>|z|)**	
*Intercept*	*-2.122*	*0.225*	*0.226*	*9.413*	*< 0.001*	***
DistForest	-0.4	0.204	0.205	1.951	0.051	.
Perimeter	1.12	0.208	0.209	5.356	< 0.001	***
Settlements	-0.302	0.197	0.197	1.531	0.126	
Ruggedness	0.566	0.205	0.206	2.75	0.006	**
DistCrops	-0.083	0.191	0.192	0.433	0.665	

## Discussion

Although not a novel phenomenon, the reporting of bear visits to human settlements in the Cantabrian Mountains has received increasing attention and has been more frequently reported in recent years. The European report *Defining*,* preventing*,* and reacting to problem bear behaviour in Europe*^25^ provides a country summary, stating for Spain: *“There are no typical habituated bears but only few cases of young bears feeding on orchards close to villages. Since habituated bears are so rare there are no common approaches to deal with them.”* Indeed, the first reports of bears in human settlements in regional media date back to 2012. This is consistent with our compilation of cases, which shows that, despite a few marginal incidents between 2009 and 2012, such visits were not become frequent until 2017 (Supplementary Table 2). It was not until 2019 that the Cantabrian Brown Bear Working Group approved the first *Protocol for Intervening with Bears in the Cantabrian Mountains*^42^, contributing to a more systematic recording and management of these events. Additionally, an increase in the awareness among residents due to social alarm and media coverage^43,44^ could influence the number of reported cases especially in these last years, with the rise and popularization of social media. This combination of demographic, administrative, and social factors makes it difficult to determine whether these events truly represent a recent behavioral phenomenon or rather a situation that has become more evident due to increased awareness and reporting.

While the inter-annual trends of these events remain complex, their spatial distribution reveals additional insights into the underlying demographic and structural factors shaping bear visits in human settlements. Bear visit events to human settlements are not evenly distributed across the range of the Cantabrian brown bear. We found 6.3 times more cases in the western part compared to the eastern part, and 3.5 times more human settlements with cases (Fig. 1; Supplementary Table 3). This discrepancy could be related to the demographic characteristics of the population. The latest published genetic study estimated 5.27 times more bears in the western population than in the eastern population^40^. A similar ratio was found in the average number of female bears with cubs in both subpopulations between 2009 and 2018, with the western subpopulation having 5.73 times more females with cubs^45^. The western population also appears to have a density 2.5 times higher than the eastern one^40^ and a presence area 1.85 times larger^31^. Additionally, a larger presence area could imply a greater number of available human settlements and, consequently, a higher number of people potentially exposed to bear presence. In this context, human density is relevant, being 10.9 people/km² in the western part and 4.9 people/km² in the eastern part^12^.

Our results showed a clear seasonal trend in bear visits to human settlements, with a peak during summer. Generally, spring (mating season) and early autumn (hyperphagia) are associated with higher numbers of bear incidents, as other authors have pointed out^25^. However, bear presence near human settlements appears to be strongly linked to the availability of anthropogenic food sources^20,46,47^. This is reflected in our findings, as 86% of the cases involved food attractants, with fruit trees, such as cherries, plums, apples, or pears, which ripen throughout summer, accounting for 55% of the documented cases. This pattern of increased visits to human settlements coinciding with the ripening of certain crops in summer has also been observed for brown bears in Hokkaido^48^ or on Vancouver Island for black bears^49^. In the urban area of Brașov, Romania, a higher incidence of human–bear conflicts during summer was linked to increased human activity and the availability of garbage dumps, garbage containers, and managed food remnants^13^. Although in the Cantabrian bear population the use of garbage as a food resource is uncommon, it should be an aspect to watch out for, because in summer the human population multiplies in these mountain villages, which generates a greater amount of waste that could encourage some individuals to learn to feed on garbage. Furthermore, the seasonal increase in human presence may also result in a higher number of potential observers, which could increase the likelihood of detecting and reporting bear visits during this period.

Alongside food conditioning, habituation to human presence is another key mechanism that appears to explain a significant portion of bear-related conflicts^27^, particularly in human-use areas where food is available^28^. However, our data show that 64% of visits to human settlement occurred at night or during twilight, a pattern similar to that reported in other European countries, where bears appeared to avoid human contact by shifting their activity towards nocturnal periods when approaching settlements^50^. Notably, only seven of the documented cases could be assumed exhibit either *little to no overt reaction to people* (habituation) or *not taking evasive or aggressive action when in the presence of people* (tolerance)^27–29^. In most of these instances, differentiating between an individual that has become habituated and one that is naturally tolerant of humans was not possible with our dataset. This is a fundamental distinction, as tolerance does not always require a prior learning process; instead of developing a reduced response through habituation (the pathways of which are discussed below), some bears may exhibit an innate predisposition to tolerate human presence^28^. Despite the lack of precise information regarding the duration of these events, most reports indicate that bears were observed in human settlements over several days. Limited human activity during nighttime and the consistent availability of easily accessible food may explain the recurrence of this behavior, particularly when hazing techniques were initiated only after several days^51^.

The demographic composition of the bears involved provides additional clues to these behavioral patterns. However, assessing the bear class involved was among the most challenging aspects of this study, with much of the data lacking precise information. This difficulty arises because many incidents were reported by local residents who often have limited training and experience in identifying the age class and sex of the animals. Nevertheless, the majority of identified cases involved young or subadult bears. These individuals, along with females with cubs, are the bear class most frequently associated with different bear incidents^52–55^. Subadult bears are less experienced in avoiding humans and face greater challenges in securing natural food resources^52^. Additionally, large bears displace young males and females with cubs from higher-quality habitats^55–58^. The displacement of young bears to avoid competition with adult males in areas of high relative density, combined with their natural dispersal behavior[^69^], increases their likelihood of using human-dominated landscapes. In our dataset, we recorded relatively few cases involving females with cubs. This could be due to instances where only a solitary individual was observed, with the cubs going unnoticed, leading to misclassification. Additionally, the literature indicates that females with cubs are displaced into more human-dominated areas during the mating season^55^, which occurs between mid-April and mid-June in the Cantabrian Mountains^59^. After this period, they tend to return to habitats similar to those used by adult males^55^. Notably, 60% of the bear visits cases in human settlements occurred from July onwards (Fig. 2a). This timing suggests that, by this point, females with cubs may have moved back to more natural areas, while subadult individuals dominate human settlement visits. These subadults are likely avoiding dominant males and are attracted to the human settlements by readily available food resources^52,53^.

From a management perspective, understanding these behavioral processes is crucial for designing effective mitigation and prevention measures. Although we could not access most reports on the interventions, we observed that the majority included hazing techniques such as shouting, fireworks, or rubber bullets. These actions align with the Cantabrian bear intervention protocol^42^ and with practices implemented in other bear populations, such as American black bears^51,60^, grizzly bears^61^, and brown bears in Europe^22^. Acoustic (e.g., shouting, fireworks) and visual methods (e.g., human presence, gestures) have been shown to be ineffective as hazing techniques, with their efficacy limited to very short-term effects^62^. In contrast, controlled pain stimuli, such as rubber bullets, have demonstrated an impact lasting for weeks after their use, though their effectiveness diminishes over the long term^22,51,63^. However, these measures can serve to deter the individual long enough from the human settlement to implement additional preventive measures that reduce the likelihood of the animal returning^51^.

A critical factor in the success of hazing techniques is the timing of its implementation, as it has been shown to be significantly more effective during the early stages of habituation or food conditioning^22,51,61,64,65^. Another key determinant appears to be the availability of anthropogenic food, with studies indicating that failing to restrict the access of bears to such food sources undermines the effectiveness of aversive conditioning^25^. However, the Cantabrian bear intervention protocol does not include references to preventive measures, such as limiting access to food sources^42^. For instance, the collection of fruit during the summer months or protecting resources with electric fences or similar preventive measures. Despite this, in the Cantabrian population cases where measures to protect food sources were implemented appear to have been successful in preventing bears from returning to human settlements. In fact, the case of a female brown bear that was moving through several villages for years, was not resolved until the almost total removal of fruit in the village, despite multiple prior interventions with hazing techniques (Junta de Castilla y León, unpublished report). This has been demonstrated in other bear populations. In Yosemite National Park, transitioning from reactive management (aversive conditioning, lethal control) to proactive strategies (limiting access to anthropogenic food, local education) led to a significant reduction in the percentage of human-derived food in black bear diets, as well as a decrease in incidents and damage^66^. This proactive approach has also proven effective with grizzly bears in Montana^47^ and Alaska^67^.

Despite the existence of a protocol, our compilation highlights the heterogeneity of the available information. Even among different administrative regions, there is no standardized recording of these events, making it challenging to classify the different types of visits and to define the different bear classes and situations. For instance, the protocol defines habituated bears as individuals that recurrently enter inhabited areas in search of accessible food resources and do not avoid humans^42^. However, this definition conflates concepts of habituation and food conditioning without precisely distinguishing between them. In comparison, definitions established in North America or at the European scale are more specific. For example, a habituated bear is defined as *“a bear that shows little to no overt reaction to people as a result of being repeatedly exposed to anthropogenic stimuli without substantial consequence”* and a food-conditioned bear as *“a bear that has learned to associate people (or the smell of people)*,* human activities*,* human-use areas*,* or food storage receptacles with anthropogenic food”* [^25^,^27–29^,^41^]. For this reason, we believe it is crucial to have a precise definition of the various scenarios observed in bear populations, particularly as intervention measures are based on these prior definitions^42^.

Beyond management and administrative considerations, the spatial and environmental characteristics of human settlements provide insights into the uneven distribution of bear visit events. At the landscape scale, we found that human settlements visited by bears were located in areas of higher habitat quality for bears and closer to the core breeding areas compared to randomly selected human settlements within the distribution range. Bears generally prefer areas with good forest cover and rugged terrain^15,18,68^, and regions near breeding cores tend to have higher bear densities^69^ which may also increase the likelihood of bear visits to nearby human settlements simply as a result of greater bear presence in the landscape[^14^]. We found that bear visits were more frequent in human settlements located in territories with higher levels of bear damage to apiculture, livestock, and fruit crops during 2012–2018. Areas with higher incidence of bear damage could be indicative of a higher availability of anthropogenic food resources, as observed in the Polish Carpathians with beehives^70^ or with livestock damages in the French Pyrenees^71^ but it can also signal learned behaviors of specific individuals. Greater availability of anthropogenic food resources may lead to individuals in the early stages of food conditioning to become more reliant on these sources, increasing their tendency to approach human settlements and potentially initiating habituation processes. It would be interesting for future studies to temporally correlate each instance of bear visit to human settlements with damages recorded in the surrounding areas during the preceding months. Furthermore, human settlements with visits are closer to each other than to random human settlements within the species’ distribution. This spatial correlation supports the hypothesis of certain conditioning and/or habituation, as it is possible that a few individuals are responsible for incidents in human settlements within their home range^46^.

Landscape-scale variables may fail to capture fine-scale factors that differentiate the likelihood of bear visits between neighbouring human settlements. Therefore, factors related to human settlement permeability, their specific characteristics, or the behavior of certain individuals in relation to their immediate surroundings should explain why bears visit some human settlements but not others within their reach. At the local scale, we observed that human settlements with bear visits are closer to forest patches than those without recorded bear visits, a pattern previously reported in studies on bear incidents^49,72^. This suggests a trade-off between accessing easily available food and the risk of leaving the protective cover of forested areas^16^. Additionally, human settlements with bear visits are located in areas with higher terrain ruggedness compared to those in the surroundings without recorded bear visits. This aligns with findings from studies on habitat selection, which concluded that bears preferentially select highly rugged areas due to the protection they provide^15,16,68,73^. Regarding the human settlement characteristics, the average model showed that human settlements with a larger perimeter have a higher probability of a bear visit. Larger human settlements might be associated with greater food availability within their boundaries (e.g., fruit trees, crops, garbage, etc.) which could reinforce the idea that bears are not entering settlements randomly, but are actively drawn to these predictable resource hotspots. Additionally, human settlements with a larger perimeter have more entry points, making it more likely for bears to encounter these larger human settlements during their regular movements compared to small human settlements. However, it is also important to consider that larger perimeters may correlate with a higher number of potential observers, thereby increasing detection probability. This could introduce an observation bias, where visits to larger settlements are more likely to be documented than those in smaller ones. Consequently, our findings reflect patterns of documented visits. Nevertheless, due to the substantial seasonal fluctuations in the number of inhabitants across villages in the Cantabrian Mountains range, we consider the perimeter to be a potentially better predictor of resource availability than human population size. While human presence varies throughout the year, resources such as fruit trees remain available, even in villages with abandoned or uninhabited houses.

Although our analyses identified several local factors associated with the likelihood of bear visits to human settlements, it is important to note that most settlements in our dataset experienced only a single event, with few repeated visits to the same location (Supplementary Table 2). This pattern suggests that these occurrences are largely circumstantial, reflecting general conditions in certain areas, such as high habitat quality, and resource availability, leading to higher bear presence, rather than consistent attraction to specific settlements. In this sense, the spatial correlation observed among villages with likely represents the influence of shared landscape characteristics rather than site fidelity by individual bears.

## Conclusions

The results of this study highlight the need to improve the data collection and the categorization of bear visit events to human settlements, enhancing transparency and data sharing, ultimately supporting more effective conflict mitigation and coexistence strategies^74^. Addressing this issue will require coordinated efforts among the entities responsible for bear management, aimed at developing standardized data collection protocols across administrative regions and linking them to intervention protocols for handling human–bear conflict situations. These protocols should be periodically updated to reflect newly observed situations in the target populations, following an adaptive management approach. Intervention measures should also be reviewed, prioritizing preventive strategies, such as the protection or even removal of trophic attractants. Our findings suggest that these attractants are a key driver of bear visits to settlements; therefore, attractant management represents a critical proactive measure that should be prioritized alongside or before the use of reactive hazing techniques. This work represents an initial step in the systematic assessment of brown bear visits to human settlements in the Cantabrian brown bear population, which could be strengthened by other methodological approaches such as telemetry or genetic studies. A better understanding of the patterns and drivers of these events would help to anticipate future conflicts, thereby promoting a balanced coexistence between humans and large carnivores.

## Supplementary Information

Below is the link to the electronic supplementary material.

Supplementary Material 1

Supplementary Material 2

## Data Availability

The datasets generated during and/or analysed during the current study are available from the corresponding author on reasonable request.
