# Supplementary material for: Patterns of brown bear (Ursus arctos) visits to human settlements provide insights for human–wildlife coexistence
Source: Sci Rep. 2026 May 17;16:22340. doi: 10.1038/s41598-026-47443-4 (PMC13377109; doi:10.1038/s41598-026-47443-4)
Supplement: Supplementary file 2 — Supplementary Material 2 [file 41598_2026_47443_MOESM2_ESM.docx]

**Title: Patterns of Brown Bear (*Ursus arctos*) Visits to Human Settlements Provide Insights for Human–Wildlife Coexistence**

Manuel Díaz-Fernández¹^,2^*, Javier Naves², Miguel de Gabriel Hernando³, Eloy Revilla²*

^1^ Fundación Oso de Asturias, Ctra. General, s/n, 33114 Proaza, Asturias, Spain (Current)

^2^ Department of Conservation Biology and Global Change, Estación Biológica de Doñana, Consejo Superior de Investigaciones Científicas (EBD-CSIC), Calle Américo Vespucio s⁄n, Sevilla 41092, Spain

^3^ Department of Biodiversity and Environmental Management, Faculty of Biological and Environmental Sciences, Universidad de León, León, Spain

Corresponding author:

Manuel Díaz-Fernández. Camín del Barreru, 143, Viella 33429, Spain.
Email: [manueldiazfernandez2@gmail.com](mailto:manueldiazfernandez2@gmail.com)

Eloy Revilla. Department of Conservation Biology and Global Change, Estación Biológica de Doñana, Consejo Superior de Investigaciones Científicas (EBD-CSIC), Calle Américo Vespucio s⁄n, Sevilla 41092, Spain

Email: revilla@ebd.csic.es
Correspondence and requests for materials should be addressed to M.D.-F.

Keywords: Brown bear (*Ursus arctos*), human-wildlife interactions, coexistence, management, population recovery

**Supplementary material**

**Supplementary Data S1.** Form used for field data collection following the initial compilation of cases provided by regional administrations.

**GENERAL DESCRIPTION OF THE CASE**

**GENERAL INFORMATION**

**Locality:**  **Contact person/interviewee:**  **Approximate date:**  **Estimated time:**  **Estimated duration:**  **Number of incidents:**

**INFORMATION RELATED TO THE BEAR**

**Type of individual:**  **Feeding:** YES ☐ NO ☐ Type: **Material damage:** YES ☐ NO ☐ Type: **Interaction with people and type:**  **Interaction with domestic animals:**  **General description of behavior:**

**INFORMATION RELATED TO PEOPLE**

**Witnesses (saw or heard the bear):** YES ☐ NO ☐ Type: **Interaction with the bear:** YES ☐ NO ☐ Type(s): **Number of people:**

**CHARACTERISTICS OF THE VILLAGE AND SURROUNDINGS**

**Approximate number of inhabitants at the time of the incident:**  **Number of inhabited houses:**  **Distance to the nearest inhabited house:**  **Distance to the nearest forest patch:**  **Forest cover in the area:**  **Access routes to the location:**

**FOOD RESOURCE AVAILABILITY**

**Fruit trees (type and approximate number):**  **Livestock (type, management, and number):**  **Garbage (type and protection):**  **Beehives (number, distance, and protection):**  **Others:**

**DETERRENT MEASURES**

**Barriers or obstacles that prevent access to resources:**

**Specific preventive measures:**  **Presence of dogs:**  **Human activity:**  **Artificial lighting (e.g., streetlights):**

**AVERSIVE MEASURES**

**In this case or nearby areas:**  **Responsible personnel:**  **Location:**

**Date and time of action:**  **Description of the measures and duration:**  **Bear behaviour and response data:**  **Possible repetitions:**


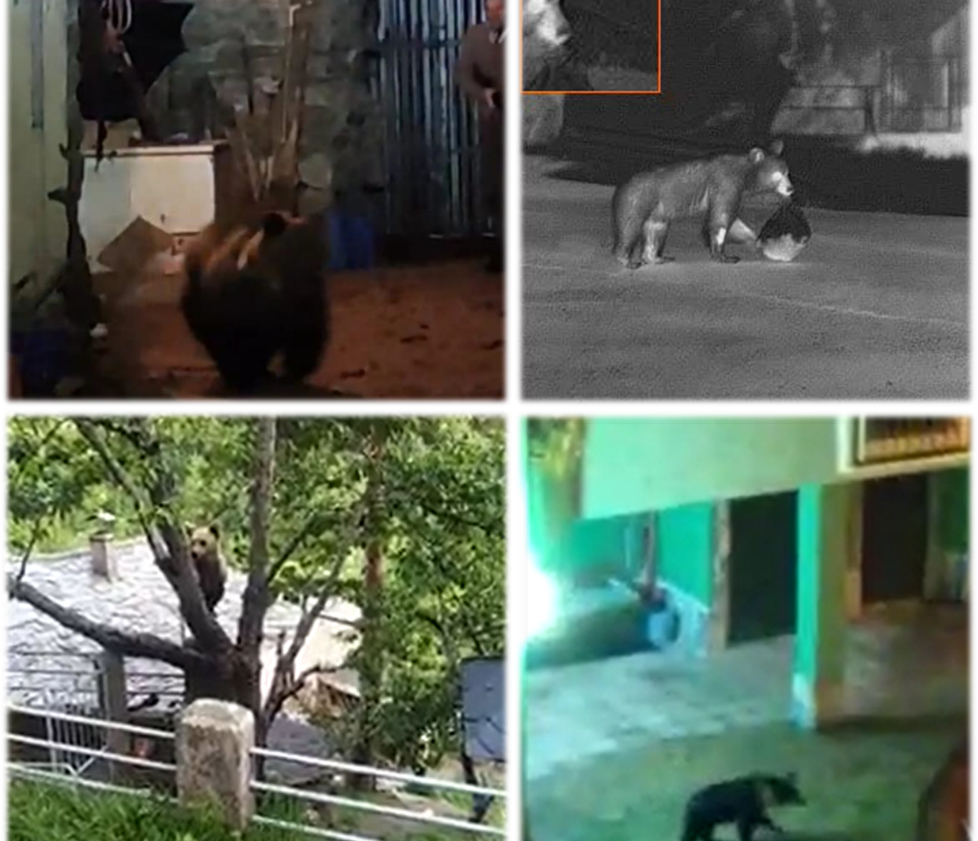


**Supplementary Fig. 1:** Different examples of photos and videos taken by some neighbors of the human settlements of some of the events described in this work. The image **above left** shows an adult female bear inside a residential enclosure, being driven away by a ranger. Photo extracted from the report from the Service of Natural Areas, Flora and Fauna of the Directorate-General for Natural Heritage and Forestry Policy regarding the negative conditioning actions carried out by the Junta de Castilla y León on the female bear “Lechuguina” (H-01R1/23-09) in application of the Intervention Protocol for Bears in the Cantabrian mountains (Junta de Castilla y León, 2023b). In the image on the **upper right** another young individual is seen looking for food in some garbage bins in the town of El Bao (Asturias). Image captured by the thermal cameras of the rangers of the Principality of Asturias. **Below left** the same individual of El Bao, feeding on a cherry tree in broad daylight. **Below right** is a bear in the building courtyard in the town of Caboalles de Abajo (province of León, Castilla y León).


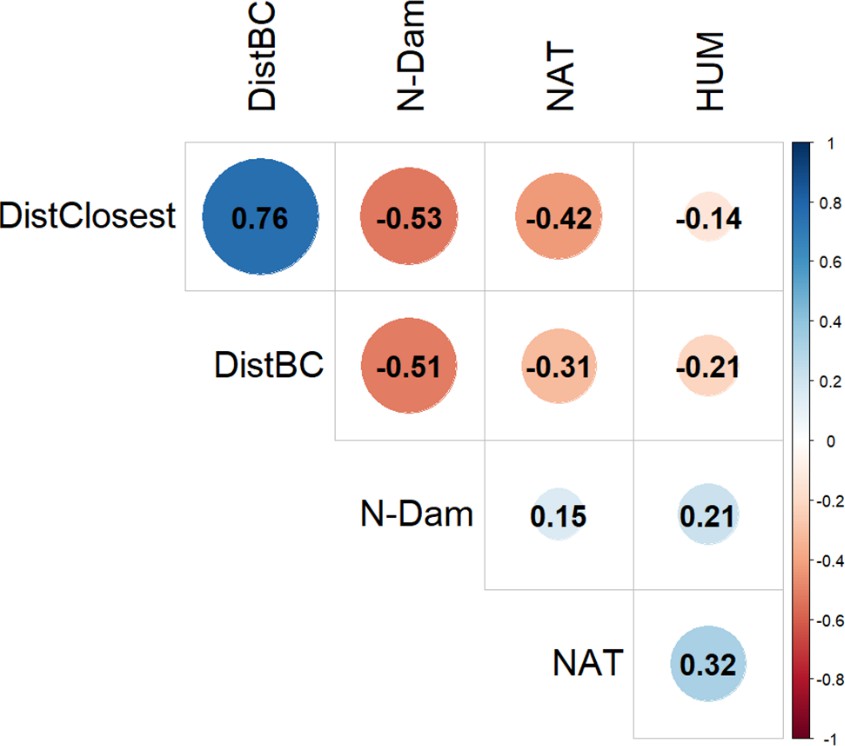


**Supplementary Fig. 2:** Correlation plot of the variables chosen as possible explanatory variables for the GLM the landscape scale analysis.

*
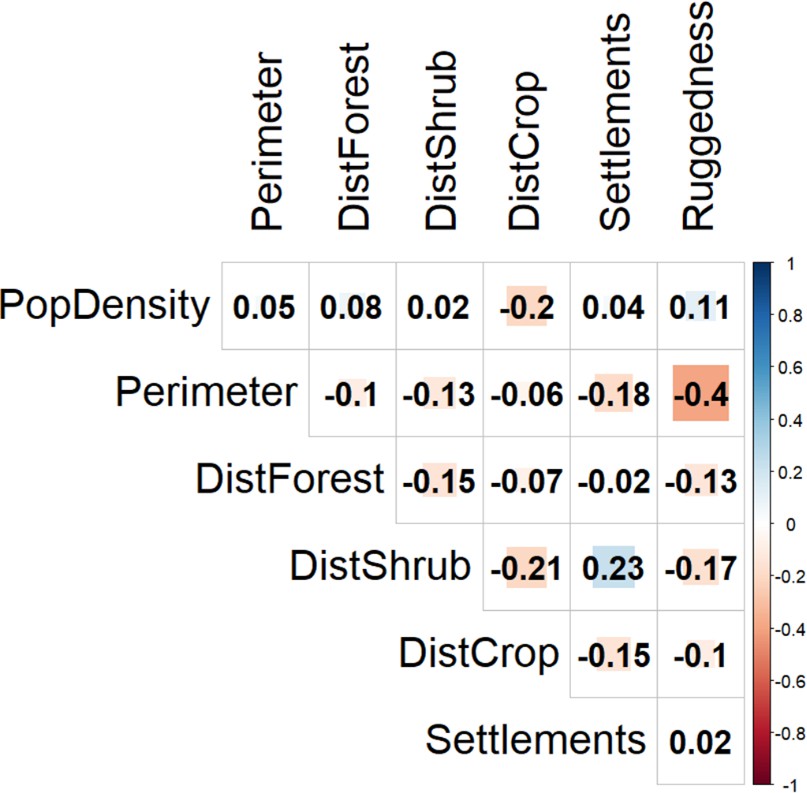
*

**Supplementary Fig. 3:** Correlation plot of the variables chosen as possible explanatory variables for the GLM the local scale analysis.

**Supplementary Table 1:** Variance Inflation Factor (VIF) values for the predictor variables included in the two binomial GLMs used in the analysis.

| **Landscape scale** | | **Local scale** | |
| --- | --- | --- | --- |
| **Predictor** | **VIF** | **Predictor** | **VIF** |
| DistClosest | 1.22 | DistForest | 1.09 |
| DistBC | 1.11 | DistShrub | 1.25 |
| N-Dam | 1.26 | DistCrop | 1.22 |
| NAT | 1.28 | PopDensity | 1.11 |
| HUM | 1.18 | Perimeter | 1.66 |
|  |  | Settlements | 1.13 |
|  |  | Ruggedness | 1.6 |

**Supplementary Table 2:** Number of bear visits recorded in each human settlement and in each of the three administrative regions with cases (Asturias, Castile and León, and Galicia) by year during the study period and as a cumulative total.

| **Region** | **2009** | **2010** | **2011** | **2012** | **2013** | **2014** | **2015** | **2016** | **2017** | **2018** | **2019** | **2020** | **2021** | **Total** |
| --- | --- | --- | --- | --- | --- | --- | --- | --- | --- | --- | --- | --- | --- | --- |
| Human settlement |  |  |  |  |  |  |  |  |  |  |  |  |  |  |
| **Asturias** | **1** |  |  | **1** |  | **1** |  | **1** |  |  | **2** | **5** | **7** | **18** |
| Castro |  |  |  |  |  | 1 |  |  |  |  |  |  |  | 1 |
| Cerredo | 1 |  |  | 1 |  |  |  |  |  |  |  |  |  | 2 |
| El Bao |  |  |  |  |  |  |  |  |  |  |  | 1 |  | 1 |
| Entrago |  |  |  |  |  |  |  |  |  |  | 1 |  |  | 1 |
| La Peral |  |  |  |  |  |  |  |  |  |  |  |  | 1 | 1 |
| Las Viñas |  |  |  |  |  |  |  |  |  |  |  | 1 |  | 1 |
| Murias de Paredes |  |  |  |  |  |  |  |  |  |  |  |  | 1 | 1 |
| Proacina |  |  |  |  |  |  |  | 1 |  |  |  |  |  | 1 |
| Proaza |  |  |  |  |  |  |  |  |  |  |  |  | 1 | 1 |
| Rebollar |  |  |  |  |  |  |  |  |  |  |  | 1 |  | 1 |
| Reguera del cabo |  |  |  |  |  |  |  |  |  |  |  |  | 1 | 1 |
| Riomolín |  |  |  |  |  |  |  |  |  |  |  | 1 |  | 1 |
| Sisterna |  |  |  |  |  |  |  |  |  |  |  |  | 2 | 2 |
| Sograndio |  |  |  |  |  |  |  |  |  |  |  |  | 1 | 1 |
| Ventanueva |  |  |  |  |  |  |  |  |  |  |  | 1 |  | 1 |
| Villarín de Cibea |  |  |  |  |  |  |  |  |  |  | 1 |  |  | 1 |
| **Cantabria** |  |  |  |  |  |  |  |  | **2** | **3** | **1** |  | **2** | **8** |
| Avellanedo |  |  |  |  |  |  |  |  |  |  |  |  | 1 | 1 |
| Caloca |  |  |  |  |  |  |  |  |  |  |  |  | 1 | 1 |
| Cambarco |  |  |  |  |  |  |  |  | 1 |  |  |  |  | 1 |
| Frama |  |  |  |  |  |  |  |  | 1 |  |  |  |  | 1 |
| Lon |  |  |  |  |  |  |  |  |  | 1 | 1 |  |  | 2 |
| Los Cos |  |  |  |  |  |  |  |  |  | 1 |  |  |  | 1 |
| Pesaguero |  |  |  |  |  |  |  |  |  | 1 |  |  |  | 1 |

| **Region** | **2009** | **2010** | **2011** | **2012** | **2013** | **2014** | **2015** | **2016** | **2017** | **2018** | **2019** | **2020** | **2021** | **Total** |
| --- | --- | --- | --- | --- | --- | --- | --- | --- | --- | --- | --- | --- | --- | --- |
| Human settlement |  |  |  |  |  |  |  |  |  |  |  |  |  |  |
| **Castilla y León** |  |  |  | **1** | **3** | **1** | **1** | **4** | **3** | **5** | **6** | **8** | **15** | **47** |
| Caboalles de Abajo |  |  |  |  |  |  |  |  | 1 | 1 | 1 | 3 |  | 6 |
| Caboalles de Arriba |  |  |  |  |  |  |  |  |  |  | 1 |  |  | 1 |
| Cuevas del Sil |  |  |  |  |  |  |  |  |  |  |  |  | 2 | 2 |
| Fresnedelo |  |  |  |  |  |  |  |  |  | 1 |  |  |  | 1 |
| Llamas de Laciana |  |  |  |  | 1 |  |  |  |  |  |  |  |  | 1 |
| Palacios del Sil |  |  |  |  |  |  |  |  |  |  |  |  | 1 | 1 |
| Polentinos |  |  |  |  |  |  |  |  |  | 1 |  |  |  | 1 |
| Rabanal de Arriba |  |  |  |  | 1 |  |  |  |  |  |  |  |  | 1 |
| Riaño |  |  |  |  |  |  |  |  | 1 |  |  |  |  | 1 |
| Rioscuro |  |  |  |  |  |  |  |  |  |  |  | 1 |  | 1 |
| Robles de Laciana |  |  |  | 1 | 1 | 1 |  |  |  |  |  |  | 1 | 1 |
| Salentinos |  |  |  |  |  |  |  |  |  |  | 1 |  |  | 1 |
| Sosas de Laciana |  |  |  |  |  |  |  |  |  |  |  | 1 |  | 1 |
| Tejedo del Sil |  |  |  |  |  |  |  |  |  |  |  |  | 1 | 1 |
| Trascastro |  |  |  |  |  |  |  |  | 1 |  |  |  |  | 1 |
| Villablino |  |  |  |  |  |  |  | 1 |  |  | 2 | 1 | 3 | 1 |
| Villager de Laciana |  |  |  |  |  |  |  |  |  |  |  |  | 5 | 5 |
| Villar de Santiago |  |  |  |  |  | 1 | 2 |  |  | 2 | 1 | 1 | 1 | 8 |
| Villarino del Sil |  |  |  |  |  |  |  |  |  |  |  |  | 1 | 1 |
| Villaseca de Laciana |  |  |  |  |  |  | 1 |  |  |  |  |  |  | 1 |
| Vivero |  |  |  |  |  |  |  |  |  |  |  | 1 |  | 1 |

**Supplementary Table 3.** Summary of bear visit to human settlements by descriptive categories. The table shows the total number of events for each subcategory and the percentage they represent out of the total.

| **Category** | **Subcategory** | **N events** | **%** |
| --- | --- | --- | --- |
| *Subpopulation* | Western | 63 | 86 |
|  | Eastern | 10 | 14 |
| *Season* | Winter-Spring (mar-may) | 9 | 12 |
|  | Summer (jun-sep) | 61 | 84 |
|  | Fall-Winter (oct-feb) | 3 | 4 |
| *Time of the day* | Night/Twilight | 47 | 64 |
|  | Day | 5 | 6.8 |
|  | Day and night | 5 | 6.8 |
|  | Unknown/Unidentified | 16 | 22 |
| *Bear class* | Young/Subadult | 27 | 37 |
|  | Adult | 12 | 16.4 |
|  | Females with cubs | 6 | 8.2 |
|  | Unknown/Unidentified | 28 | 38.4 |
| *Trophic attractant* | Fruit trees | 40 | 55 |
|  | Crops | 14 | 19.18 |
|  | Livestock | 8 | 10.96 |
|  | Garbage | 8 | 10.96 |
|  | Domestic animal feed | 8 | 10.96 |
|  | Others | 5 | 6.85 |
|  | Some attractants | 22 | 30.14 |
| *Duration* | >1 day | 39 | 53 |
|  | 1 day | 16 | 22 |
|  | Unknown/Unidentified | 18 | 25 |
| *Human-bear interaction* | Yes | 29 | 40 |
|  | Tolerance of human presence | 7 | 9.5 |
|  | Unknown | 44 | 60 |
| *Interventions* | Yes | 26 | 36 |
|  | Hazing | 16 | 22 |
|  | Only monitoring | 5 | 7 |
|  | Preventive measures | 3 | 4 |
|  | Unidentified | 2 | 3 |
|  | Unknown | 47 | 64 |

**Supplementary Table 4:** Best generalized linear regression models (ΔAICc < 2) to explain the probability of bear visit to a human settlement related to landscape scale factors ranked by the ΔAICc values.

| **Model** | **Degrees of freedom** | **AIC** | **ΔAICc** | **Weight** | **Log-Likelihood** |
| --- | --- | --- | --- | --- | --- |
| **DistBC + NAT + N-Dam +**  **DistClosest** | **4** | **35.09** | **0** | **0.1663** | **-13.5** |
| DistBC + DistClosest | 3 | 35.2 | 0.11 | 0.1577 | -14.57 |
| DistBC + NAT | 4 | 35.22 | 0.12 | 0.1563 | -13.57 |
| DistBC + NAT + N-Dam +  DistClosest | 5 | 35.8 | 0.71 | 0.1169 | -12.83 |
| NAT + N-Dam + DistClosest | 4 | 35.98 | 0.89 | 0.1066 | -13.95 |
| NAT + DistClosest | 3 | 36.36 | 1.27 | 0.0881 | -15.16 |
| DistBC + N-Dam | 4 | 36.54 | 1.45 | 0.0805 | -14.23 |
| DistBC + NAT + N-Dam + HUM | 5 | 36.97 | 1.88 | 0.065 | -13.42 |
| DistBC + DistClosest + HUM | 4 | 37.04 | 1.95 | 0.0628 | -14.48 |

**Supplementary Table 5:** Best generalized linear regression models (ΔAICc < 2) to explain the probability of bear visit to a human settlement related to local scale factors ranked by the ΔAICc values.

| **Model** | **Degrees of freedom** | **AIC** | **ΔAICc** | **Weight** | **Log- Likelihood** |
| --- | --- | --- | --- | --- | --- |
| **DistForest + Perimeter + Settlements + Ruggedness** | **5** | **202.9** | **0** | **0.451** | **-96.353** |
| DistForest + Perimeter + Ruggedness | 6 | 203.3 | 0.37 | 0.375 | -97.574 |
| DistForest + Perimeter + Settlements + Ruggedness + DistCrops | 6 | 204.8 | 1.90 | 0.174 | -96.258 |
